# REVIRAL: roadmap for the elimination of viral hepatitis in Latin America

**DOI:** 10.3389/fpubh.2025.1527704

**Published:** 2025-05-16

**Authors:** Javier Crespo, Joaquín Cabezas, Graciela Castro-Narro, Hugo Cheinquer, Fernando Contreras, Nelia Hernandez, Christie Perelló, Ezequiel Ridruejo, José Luís Calleja

**Affiliations:** ^1^Gastroenterology and Hepatology Department, University Hospital Marqués de Valdecilla, Santander, Spain; ^2^Research Institute Valdecilla (IDIVAL in Spanish), Santander, Spain; ^3^School of Medicine, University of Cantabria, Santander, Spain; ^4^Gastroenterology and Hepatology Department, Instituto Nacional de Ciencias Médicas y Nutrición Salvador Zubirán, Ciudad de México, Mexico; ^5^Gastroenterology and Hepatology Department, Hospital de Clínicas de Porto Alegre, Universidade Federal do Rio Grande do Sul, Porto Alegre, Brazil; ^6^Centro de Gastroenterología Avanzada de Santo Domingo, Santo Domingo, Dominican Republic; ^7^Gastroenterology and Hepatology Department, Hospital de Clínicas, Facultad de Medicina, Universidad de la Republica, Montevideo, Uruguay; ^8^Gastroenterology and Hepatology Department, Puerta de Hierro University Hospital, Madrid, Spain; ^9^Puerta de Hierro Majadahonda Research Institute (IDIPHIM), Madrid, Spain; ^10^School of Medicine, Autonomous University of Madrid, Madrid, Spain; ^11^Hepatology Section, Department of Medicine, Centro de Educación Médica e Investigaciones Clínicas Norberto Quirno “CEMIC”, Buenos Aires, Argentina

**Keywords:** viral hepatitis, elimination, epidemiology, models of care, linkage-to-care

## Abstract

The REVIRAL project proposes a comprehensive and tailored strategy for the elimination of viral hepatitis in Latin America, drawing inspiration from successful models in other countries. Despite global advances, the region faces specific barriers, including the lack of universal screening programs, disparities in access to direct-acting antiviral treatments, the presence of fragmented health systems, and the social stigma associated with these infections. These factors hinder progress towards elimination, necessitating collaborative responses tailored to the epidemiological and socioeconomic realities of each country. The project’s structure is organized into three fundamental phases: The first phase involves an initial assessment, where the epidemiological situation of each country will be analyzed, specifically the prevalence and incidence data of viral hepatitis and the response capacity of each country’s health systems. This phase will also identify specific barriers, allowing interventions to be adjusted to local needs. The second phase will focus on the implementation of intervention programs, promoting universal screening, strengthening healthcare personnel training, and implementing health models that enhance linkage-to-care. The third phase will concentrate on monitoring and evaluating results through a global elimination marker, designed to measure and compare progress across countries. The success of REVIRAL will largely depend on the commitment of local governments and international cooperation. If implemented correctly, the project could transform the public health response to viral hepatitis in Latin America and serve as a replicable model for other regions with similar challenges advancing toward the WHO’s global elimination goals set for 2030.

## Introduction

The World Health Organization (WHO) has identified the elimination of viral hepatitis as a major global public health goal (1). In 2016, ambitious targets were set to eradicate these infections as a public health threat by the year 2030. This global action plan aims to reduce the incidence of new chronic hepatitis infections by 90% and decrease hepatitis-related mortality by 65% (2). To achieve these objectives, the WHO has recommended early detection and treatment for at least 80% of eligible patients, employing direct-acting antivirals (DAAs) for HCV and nucleotide analogs for HBV management. Despite advances in the diagnosis and treatment of viral hepatitis worldwide, significant challenges remain. In high-prevalence areas with constrained health systems, diagnostic and therapeutic coverage is limited. For HCV, DAAs have revolutionized treatment; however, the availability of these treatments is not equitable, especially in low-and middle-income regions where access is restricted. The absence of universal screening programs across various populations hinders early case identification and complicates effective infection control. Regarding HBV, control efforts remain complex, as it persists as an endemic disease in several regions, particularly in Latin America and Africa. HBV coinfection with hepatitis D virus (3) (HDV) in certain areas has increased the incidence of severe complications such as cirrhosis and hepatocellular carcinoma. This situation underscores the importance of implementing effective vaccination strategies and expanding control and elimination efforts, focusing on the use of specific nucleotide analogs for HBV management and improving access to diagnosis and treatment for HBV-HDV coinfection.

Despite growing global efforts, there is no integrated, multicountry roadmap in Latin America specifically tailored to the region’s structural disparities and social challenges in eliminating hepatitis B, C, and D. Previous publications have addressed individual country strategies or theoretical frameworks (4–6), but none propose a unified, operational, and phased implementation model that integrates micro-elimination, standardized indicators, and continuous monitoring at the regional level. REVIRAL seeks to fill this gap by providing a practical and adaptable tool aligned with WHO and Panamerican Health Organization (PAHO) goals.

Latin America presents a heterogeneous landscape regarding the implementation of strategies for the elimination of hepatitis C (4). As of 2023, 78.9% of countries in the region have adopted a national plan for HCV elimination. However, only 21.1% of these countries, including Argentina, Colombia, and Mexico, have implemented certain local universal screening programs that do not cover the general population. One of the countries with the highest viral hepatitis burden (measured in disability-adjusted life years) and ranked among the top 20 accounting for more than 75% of the global viral hepatitis burden is Brazil. Key data from Brazil illustrate this issue (7). Despite notable progress, between 2000 and 2022, Brazil’s National Health System reported 276,646 new HBV diagnoses and 298,738 new HCV diagnoses, with a decrease in both HBV and HCV incidence (8–11). Although both HBV and HCV treatments in Brazil are publicly funded, only 25% of people requiring HBV treatment are treated (approximately 25,000) (11), and HCV treatment rates significantly declined in 2020 due to COVID-19. According to Brazil’s National HCV Elimination Plan, 154,811 people have received HCV treatment since October 2015, representing 27% of its elimination target for 2030. A coordinated response in all countries is desirable; however, in nations like Brazil, such a response is imperative to achieve elimination goals across LATAM. The lack of systematic screening in most countries significantly hampers early case identification, leading to delays in treatment initiation and suboptimal disease control including late-stage diagnosis (5), particularly in asymptomatic patients. Access to direct-acting antivirals (DAAs) reveals considerable disparity across the region. Although 78.9% of Latin American countries theoretically offer universal access to these drugs, access is restricted in nations such as Cuba, Ecuador, and Venezuela, affecting approximately 37.9% of the population. In many countries with purported universal access, barriers are so significant that only a minority of the population ultimately receives treatment. This discrepancy between theoretical access and practical availability underscores inequities in treatment, affecting cure rates and reducing the virus transmission rate in the general population. Furthermore, only 36.8% of countries have access to generic versions of DAAs, which heightens economic barriers in resource-limited settings. On the other hand, referral and follow-up systems exhibit significant variation among countries. While 63.2% of nations have implemented direct referral systems for patients diagnosed with active HCV infection, only 36.8% have alert systems that automatically notify treating physicians when a patient tests positive. Additionally, only 15.8% of countries (Argentina, Brazil, Colombia) have implemented retrospective strategies to track patients lost to follow-up, which is essential to prevent infection spread and disease progression in individuals not receiving timely treatment. For HBV and HDV, diagnostic and treatment coverage remains insufficient in most countries in the region. Although some countries have advanced in implementing HCV screening programs, systematic detection of HBV, and particularly HDV, has received less priority. This lack of focus limits comprehensive viral hepatitis control, hindering adequate management of HBV-HDV coinfection, which is responsible for increased morbidity and mortality. To enhance the regional response, countries need to not only strengthen HBV and HDV screening programs but also optimize vaccination strategies. Although some countries include neonatal HBV vaccination in their national schedules, these programs do not always adequately reach high-risk populations or even complete the 3 doses schedule, perpetuating transmission and associated complications. Including accurate data on HBV and HDV prevalence, as well as a rigorous evaluation of vaccination and treatment programs, is essential to advance viral hepatitis elimination in Latin America (6).

## Main barriers to viral hepatitis elimination in Latin America

In Latin America, the elimination of viral hepatitis faces structural, economic, and social barriers (12) that limit the health systems’ response capacity. These barriers highlight the need to strengthen public health policies in the region, promoting equitable access to both diagnostics and treatment (5, 12). One of the main barriers is the fragmentation of health systems. In many countries, there is a significant division between public and private healthcare services, creating disparities in medical care, particularly in rural or underdeveloped areas. This division limits equitable access to healthcare services, adversely affecting the capacity to implement large-scale screening and treatment programs. Despite international recommendations promoting screening for at-risk populations, systematic detection programs for viral hepatitis remain insufficient in the region. Only a limited number of countries have implemented adequate measures for early infection identification, compromising the ability to intervene effectively and promptly. This lack of screening programs widens the gap between diagnosis and treatment. In addition, significant disparities in access to direct-acting antivirals (DAAs) persist. Although most countries in the region have incorporated DAAs into their health systems, high costs and limited availability of generic versions hinder equitable access to these treatments, especially in nations with weaker economies and less developed health systems. In many countries, viral hepatitis treatment is not cover for private insurance leaving all the burden to the public healthcare system. This perpetuates inequality in treatment access and reduces opportunities to achieve uniform cure rates throughout the region. Another key issue is the inadequacy of notification and referral systems for active HCV infection cases. A significant number of countries lack automated notification mechanisms to inform treating physicians about positive results, slowing down the referral and treatment process. Without these tools, many patients fall outside follow-up programs, compromising both infection control and elimination efforts. In terms of case follow-up, few countries have developed effective methods to locate and treat previously diagnosed patients who are lost within the healthcare system. The lack of adequate follow-up increases the risk of disease progression in these individuals and reduces cure rates at a population level.

For HBV and HDV, the situation is even more complex. The fragmentation of health systems and public policies also limits access to both treatment and vaccination. The lack of accessible diagnostic tests and the restricted availability of therapies for HDV hinder a comprehensive micro-elimination approach, perpetuating transmission and negatively impacting public health. Additionally, limited awareness of HBV-HDV coinfection among healthcare professionals and the general population constitutes an additional barrier that must be addressed through education and awareness programs. Finally, stigma associated with hepatitis infections, particularly in the case of HCV, remains a significant barrier to diagnosis and treatment. Fear of discrimination or social rejection discourages many people from seeking medical care, delaying intervention and contributing to ongoing virus transmission. Combating this stigma through public education and awareness campaigns is crucial to facilitating access to healthcare services and improving clinical outcomes across the region.

Regarding HBV, one of the most significant barriers remains the low vaccination coverage among high-risk populations and the limited availability of rapid diagnostic tests. The high complexity of the diagnostic process also hampers effective patient treatment. For HDV, barriers include limited access to specialized diagnostic tests, the absence of systematic reflex testing, and the restricted availability of effective antiviral therapies (13, 14). The high rate of HBV-HDV coinfection in certain groups, such as people who inject drugs, represents an additional challenge, as it requires specific and coordinated therapeutic approaches to reduce disease progression and improve clinical outcomes in these vulnerable populations.

## Strategies to overcome these barriers to elimination

The success in eliminating viral hepatitis (particularly HCV) in more advanced countries has relied on the implementation of five fundamental strategies (7) that have proven decisive in advancing toward disease elimination (15, 16). First, the existence of national strategic plans (17). These plans, aligned with WHO guidelines (1), not only focus on increasing diagnostic and treatment coverage but also implement an integrated approach encompassing multiple levels of the healthcare system. Second, universal, equitable, and financially unrestricted access to antiviral treatment is another cornerstone of these successful models (18), facilitating rapid and effective access to treatment regardless of economic status or geographic location and including treatment decentralization. Adequate micro-elimination policies targeting high-risk groups, such as incarcerated individuals and people who inject drugs, have significantly contributed to disease control in the broader population. Undoubtedly, information and awareness campaigns have encouraged a greater number of people to actively seek diagnosis and treatment and foster broader acceptance of necessary healthcare interventions. Eliminating social stigma is a key element for effective disease control. Finally, strengthening surveillance and follow-up systems has been essential to the success of these countries. The implementation of robust case monitoring and notification systems has enabled continuous evaluation of progress, rapid identification of areas requiring improvement, and ensured that corrective actions are sustainably implemented.

To effectively adapt viral hepatitis elimination strategies in Latin America, it is essential to deepen the understanding of each country’s epidemiological situation and the capacity of its health systems. This will allow for more specific and targeted interventions based on local needs. Additionally, establishing a global elimination marker is crucial to facilitate progress measurement and standardize objectives at a regional level. It is imperative to enhance the understanding of viral hepatitis prevalence and distribution through detailed studies in each nation, providing precise information on epidemiological characteristics and areas of greatest need. This will enable the design of intervention programs tailored to the particularities of each population, prioritizing resources efficiently.

The definition of a global elimination marker is key to objectively evaluating each country’s progress (19). This set of indicators should allow for standardized comparison at the regional level, promoting cohesion in elimination efforts. Furthermore, it would facilitate the exchange of best practices and the real-time adjustment of strategies, improving coordination among different countries. Focusing on both micro-and macro-elimination policies should be a priority to address vulnerable populations and the general population alike. Efforts should concentrate on high-risk groups, such as people who inject drugs, without neglecting the implementation of public health policies that reach the broader population. This combined approach would ensure that transmission hotspots within more vulnerable subgroups are not perpetuated, while advancing elimination across the entire region.

Two major aspects, which affect not only Latin America but also other regions worldwide, must be implemented as efficiently and rapidly as possible: the simplification of diagnosis and treatment. Simplified diagnostics and point-of-care rapid testing are essential to addressing elimination and have long been available for both HBV and HCV (20, 21) including HCV PCR (22). A greater effort is essential to develop and use point-of-care technologies that become the standard of care. Furthermore, simplifying the treatment of viral hepatitis remains a priority to enable rapid service expansion. There have been advances with HCV (23); however, the same is not yet true for HBV treatment, which still requires HBeAg, anti-HBe, HBV-DNA, and liver fibrosis evaluation (24, 25). The complexity of care complicates treatment for these patients, and its simplification is already being addressed in the new WHO recommendations (26). To overcome economic barriers limiting treatment access, it is essential to facilitate the availability of affordable diagnostics and treatments (27). Through price negotiations and the inclusion of DAAs in national health programs, it is possible to significantly reduce financial barriers that prevent equitable access to treatment (28). These actions would not only improve cure rates but also reduce the disease burden on regional health systems, making the elimination effort more sustainable in the long term.

## REVIRAL project: roadmap for the elimination of viral hepatitis in Latin America

The REVIRAL project aims to establish a clear and specific pathway for the elimination of viral hepatitis in Latin America, addressing the region’s unique epidemiological, structural, and social needs. Unlike generalized strategies, REVIRAL focuses on developing a regional response that tackles specific barriers in diagnosis, treatment, and follow-up, while adapting to the socioeconomic realities of each country. This project seeks to coordinate efforts at a regional level, promoting multinational collaboration to share best practices, public health policies, and resources, thereby enhancing the effectiveness of elimination programs in Latin America. REVIRAL aligns with the WHO’s 2030 targets (19) and proposes to establish a set of global elimination indicators that can be monitored and adjusted according to each country’s progress, providing a model for continuous and adaptable evaluation.

## Specific objectives of the REVIRAL project

**Figure 1:**
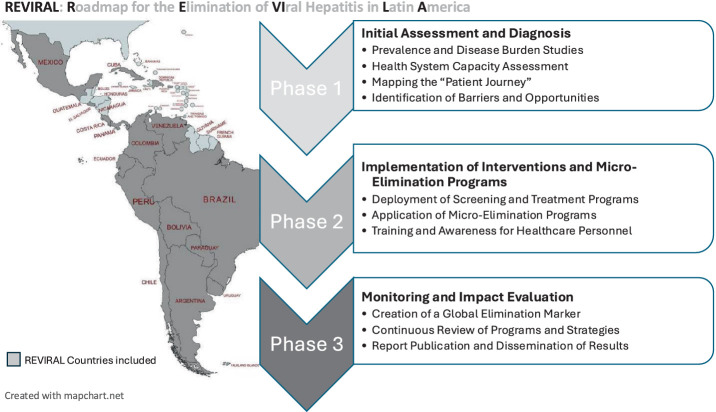
REVIRAL project: country components and summary design. Created with mapchart.net, licensed under CC BY 4.0.

The primary goal of the REVIRAL project is the elimination of viral hepatitis in Latin America through a collaborative approach tailored to each country’s specific needs. To achieve this goal, the project aims to develop strategies to accomplish the following specific objectives (Figure 1):

1. Increase diagnostic coverage for hepatitis B, C, and D: Improve access and screening coverage through the implementation of universal and targeted detection programs (macro-and micro-elimination), specifically addressing high-risk populations, including people who use drugs, incarcerated individuals, and migrants.
2. Ensure equitable access to direct-acting antiviral treatments for HCV, nucleotide analogs for HBV, and, in rare cases, Bulevirtide for HDV: Advocate for the inclusion of these drugs in the public health systems of all participating countries, facilitating their distribution at affordable, and, whenever possible, free of charge, prioritizing patients with the greatest vulnerability.
3. Reduce stigma and cultural barriers: Promote public awareness and health education campaigns to facilitate the inclusion of stigmatized and hard-to-reach populations in elimination programs with potential integration of HIV and syphilis screening through comprehensive diagnostic strategies when feasible.
4. Implement micro-elimination initiatives in specific settings, such as prisons, homeless shelters, and indigenous communities, adapting resources and care protocols to the particular needs of each population.
5. Develop a continuous monitoring and evaluation system: Create a follow-up and monitoring platform to record progress in each country, using standardized indicators fed by data collected in each phase of the project.

## Implementation and monitoring phases

The implementation of the REVIRAL project will be developed in three main phases, designed to allow an orderly and measurable progression towards the elimination goals.

### Phase 1: initial assessment and diagnosis

The first phase will focus on data collection and analysis to establish a baseline for the hepatitis situation in each country, encompassing both the prevalence of these infections and the capacity of health systems to effectively meet service demands. During this phase, the following will be conducted:

- Prevalence and Disease Burden Studies: Using national and regional epidemiological data, a detailed view of the burden of hepatitis B, C, and D in each country will be obtained. Additionally, field studies will be conducted in areas with limited data availability to ensure accurate representation.
- Health System Capacity Assessment: The resources and infrastructure available in each country will be analyzed, identifying gaps in patient diagnosis, treatment, and follow-up. This will allow for the identification of areas for improvement to strengthen the response capacity of each health system.
- Mapping the “Patient Journey”: The typical patient pathway from diagnosis to treatment and follow-up will be studied. This analysis will include diagnostic test availability, waiting times, retention rates in the system, and social challenges that may interfere with treatment so it can be design patient care cascade (29).
- Identification of Barriers, Norms, and Drivers: Based on the findings from the initial assessment, the specific barriers, emerging or needed norms (e.g., standardized referral systems or test-and-treat protocols, management guidelines (3, 30–32)), and existing or potential drivers (e.g., community-based health programs, engaged professional societies) for each country will be defined. This will support the development of a personalized action plan that reflects local contexts and mobilizes positive forces alongside removing limitations (19, 29). This first phase is crucial for adjusting the goals and activities of REVIRAL, ensuring that the elimination strategy is both effective and sustainable over time.

### Phase 2: implementation of interventions and micro-elimination programs

In this phase, REVIRAL will focus on deploying intervention programs encompassing screening to treatment, adapted to local realities and aimed at achieving elimination in high-risk populations.

- Deployment of Screening and Treatment Programs: Based on the information gathered in the initial phase, screening campaigns will be conducted in clinics and communities, prioritizing high-prevalence areas and groups. Systematic screening practices will be promoted in hospitals, health centers, and mobile clinics to achieve extended diagnostic coverage.
- Application of Micro-Elimination Programs: REVIRAL will implement specific programs in high-prevalence settings such as prisons, homeless shelters, and indigenous communities. These programs will be tailored to the specific conditions of each group and will be accompanied by protocols for the administration of treatments, known as test-and-treat strategies (33).
- Training and Awareness for Healthcare Personnel: With support from the health authorities of each country, REVIRAL will offer training workshops to ensure that healthcare personnel are prepared to manage and treat viral hepatitis. These workshops will include training on the use of DAAs, techniques to reduce stigma, and follow-up protocols (34). This phase will allow progress in viral hepatitis elimination through a micro-elimination approach in key populations, ensuring that resources are efficiently allocated to groups with higher vulnerability and transmission risk.

### Phase 3: monitoring and impact evaluation

The third phase will focus on the surveillance and collection of results, as well as impact evaluation to ensure progress towards elimination. This final phase of REVIRAL will emphasize continuous monitoring of results and impact evaluation of the implemented interventions, through an elimination indicator system that will facilitate comparison of progress across the region. The following actions will be developed:

- Creation of a Global Elimination Marker: As a central component of REVIRAL, an elimination marker will be established, including metrics to evaluate progress in screening, diagnosis, access to treatment, and morbidity and mortality reduction. This marker will enable standardized comparison between countries and serve as a reference for real-time strategy adjustment.
- Continuous Review of Programs and Strategies: The data obtained will be used to make adjustments to programs as necessary, ensuring the project’s sustainability and adaptability over the long term. Periodic reports will allow identification of areas needing improvement and alignment of efforts with global elimination goals.
- Report Publication and Dissemination of Results: To ensure transparency and foster collaboration, REVIRAL will generate regular reports to be shared with local partners, health authorities, and the international community. The dissemination of results will also allow for the exchange of best practices and the strengthening of regional cooperation. The monitoring phase ensures the long-term sustainability and effectiveness of the project, enabling adaptation of public health policies and decisions based on observed results, optimizing elimination efforts throughout Latin America.

## Discussion

The elimination of viral hepatitis in Latin America presents a complex challenge due to the multiple economic, structural, and social barriers present in the region. Unlike regions where health systems have more robust infrastructure and equitable treatment coverage, some Latin American countries face a lack of coordination in health systems, disparities in access to diagnostics and treatments, and significant levels of stigmatization that limit patients’ access to adequate care. International experience shows that micro-elimination strategies, combined with strong community involvement, are effective in addressing hepatitis in hard-to-reach subgroups and, collectively, impact global elimination goals (35, 36). For example, HCV elimination programs in prisons and rehabilitation centers in some European countries and Australia have achieved a significant reduction in hepatitis transmission. This strategy has been adapted in the REVIRAL project to focus on high-risk populations in Latin America. REVIRAL’s micro-elimination programs are designed to reduce the viral burden in marginalized communities, thereby reducing transmission in the general population. Furthermore, the focus on the “patient journey” through the healthcare system—another innovative component of REVIRAL—will help identify critical points where access to diagnosis or treatment may be disrupted. This is particularly relevant in Latin America, where health systems face challenges in terms of continuity of care, making early detection and proper follow-up of patients difficult. This fragmentation not only delays care but also limits the capacity to integrate hepatitis control into broader health system functions, reinforcing the need for standardized protocols and regional cooperation.

Another relevant aspect of the project is the creation of a global elimination marker. This component allows for standardized tracking of progress in each country, something especially crucial in a context where health systems are diverse and face varying degrees of limitations. By offering a uniform methodology, the marker facilitates the identification of successful practices and the replication of effective policies throughout the region.

While the REVIRAL project thoroughly identifies and addresses key barriers to hepatitis elimination, we acknowledge the importance of adopting a more symmetrical conceptual framework. In this regard, we propose complementing the current approach with two additional analytical dimensions: (1) the establishment of norms, understood as institutionalized behaviors and policies (e.g., universal screening protocols, real-time data reporting, or reflex testing); and (2) the activation of drivers, which include cultural facilitators, committed stakeholders, and structural enablers. This broader perspective allows us not only to remove obstacles but also to mobilize positive forces already present within local systems. For instance, instead of focusing solely on vaccine hesitancy (a barrier), we can also foster vaccination urgency by engaging community leaders or integrating hepatitis testing into routine primary care. Similarly, continuous monitoring can be framed not only as a technical necessity but as a normative expectation across national programs (19, 27).

Although the REVIRAL project is focused on the elimination of hepatitis B, C, and D, it is crucial to recognize the opportunity to integrate testing for other bloodborne infections—namely HIV and syphilis—into its operational activities. These infections share transmission routes and disproportionately affect similar at-risk populations, including people who inject drugs (PWID), incarcerated individuals, and sex workers. Importantly, joint screening using a single blood sample is feasible and cost-effective, especially in resource-limited settings.

Experiences such as the Crivalvir-Focus (37) program in Europe have demonstrated that excluding HIV or syphilis testing from hepatitis-focused interventions may result in missed diagnoses, which delays linkage to care. Moreover, UNAIDS has proposed the 95–95–95-0 target for 2030: 95% of people living with HIV diagnosed, 95% on sustained antiretroviral therapy, 95% with viral suppression, and 0% stigma and discrimination. These targets align closely with the elimination logic of REVIRAL. From a public health perspective, the implementation of REVIRAL at national or subnational levels should seek operational convergence with HIV and syphilis programs, especially in micro-elimination settings. This approach would maximize impact and cost-effectiveness, and respond more holistically to the epidemiological burden in Latin America (38, 39).

In the framework of the REVIRAL project, we are fully committed to implementing best clinical practices for the screening, diagnosis, follow-up, and treatment of viral hepatitis. Among these, we will unequivocally promote the concept of comprehensive diagnosis, understood as the systematic screening for all major viral hepatitis and HIV whenever any one of these infections is detected, as well as the routine screening for viral hepatitis and HIV in all individuals diagnosed with sexually transmitted infections. We are firmly convinced that this integrated diagnostic strategy will not only enhance the detection and management of viral hepatitis but will also lead to improved diagnosis of HIV and other sexually transmitted infections, thereby contributing to a broader public health impact.

However, the success of REVIRAL will largely depend on cooperation between health authorities, service providers, and civil society. Previous experiences in the region have shown that public health programs are only sustainable when supported by multiple sectors and when health policies are adapted to local socioeconomic realities. To maximize the impact of REVIRAL, it will be crucial to ensure active financial and political commitment from governments and to foster ongoing collaboration among countries. The project’s success will also depend on its ability to comprehensively address the elimination of all three viral hepatitis types. While HCV has received more attention due to advances in its treatment, HBV and HDV require additional strategies, such as universal HBV vaccination and the development of specific antiviral therapies for HDV. Integrating these approaches will enable REVIRAL to advance toward the total elimination of viral hepatitis in Latin America, consolidating its impact on public health and bringing the region closer to the global elimination goals set by the WHO.

## Study limitations and future directions

Although REVIRAL proposes a regional roadmap for elimination, the availability and granularity of data vary significantly across countries. In some cases, surveillance systems are incomplete or inconsistent, particularly regarding HDV or retrospective tracking systems. The reported is based on the latest data from ministries and regional reports. These limitations underscore the need for investment in regional data systems and caution in interpreting regional comparisons.

## Conclusion

The REVIRAL project represents an innovative and flexible approach designed to drive viral hepatitis elimination in Latin America. Based on the progress achieved in countries where viral hepatitis elimination has significantly advanced, REVIRAL offers a phased implementation model adapted to each country’s specific characteristics, integrating strategies for detection, treatment, stigma reduction, and micro-elimination in high-risk groups.

The phases of initial assessment, specific intervention implementation, and continuous monitoring ensure that the project aligns with the WHO’s 2030 goals while adapting to local resources and specificities. The creation of a global elimination marker provides a standardized tool to evaluate progress in real-time, facilitating the identification of areas for improvement and the adoption of best practices at a regional level.

To maximize impact, REVIRAL implementation should be adapted to the specific capacities and gaps of each country. For instance, countries like Argentina and Brazil, which already have national elimination plans, could prioritize expansion of retrospective follow-up and inclusion of HDV screening. Others, such as Ecuador or Venezuela, may first require basic infrastructure for diagnostic and treatment access. Practical adoption of global indicators and flexible micro-elimination strategies should guide national adaptation, ensuring alignment with WHO and PAHO goals in a resource-sensitive manner.

For Latin America, a successful implementation of REVIRAL could mark a milestone in reducing the burden of viral hepatitis and improving overall public health. However, the project’s sustainability will largely depend on regional collaboration and the active commitment of health authorities and social actors. In summary, REVIRAL emerges as an effective and scalable tool to advance toward the elimination of viral hepatitis in Latin America, providing a replicable model for other regions facing similar challenges. Additionally, by incorporating HBV and HDV along with HCV, this approach ensures a comprehensive elimination of viral hepatitis, maximizing its public health impact and paving the way to achieving the WHO goals in an equitable and sustainable manner.

## Data Availability

The datasets presented in this study can be found in online repositories. The names of the repository/repositories and accession number(s) can be found in the article.
