# Supplementary material for: REVIRAL: roadmap for the elimination of viral hepatitis in Latin America
Source: Front Public Health. 2025 May 16;13:1527704. doi: 10.3389/fpubh.2025.1527704 (PMC12123641; doi:10.3389/fpubh.2025.1527704)
Supplement: Supplementary file 1 [file Table_1.docx]

Supplementary Material

# REVIRAL project, study group:

| **Coordinator leaders** | | |
| --- | --- | --- |
| Crespo García, Javier |  |  |
| Calleja Panero, José Luís |  |  |
| Ridruejo, Ezequiel |  |  |
| **Steering committee** | | |
| Cabezas, Joaquin |  |  |
| Castro-Narro, Graciela |  |  |
| Cheinquer, Hugo |  |  |
| Perello, Christie |  |  |
| Contreras, Fernando |  |  |
| Crespo García, Javier |  |  |
| Calleja Panero, José Luís |  |  |
| Ridruejo, Ezequiel |  |  |
| Hernández, Nelia |  |  |
| **Local investigators** | | |
| **Argentina** | | |
| Mendizabal, Manuel |  |  |
| Cairo, Fernando |  |  |
| Ridruejo, Ezequiel |  |  |
| **Brasil** | | |
| Pessôa, Mário Guimarães |  |  |
| Eduardo, Emerim |  |  |
| Cheinquer, Hugo |  |  |
| **Bolivia** | | |
| Guerra, Patricia |  |  |
| **Chile** | | |
| Zapata, Rodrigo |  |  |
| Soza, Alejandro |  |  |
| Nazal, Leyla |  |  |
| **Colombia** | | |
| Beltrán, Oscar |  |  |
| Hernández, Javier |  |  |
| Garzón, Martin |  |  |
| **Costa Rica** | | |
| Coste, Pablo |  |  |
| Alvarado Salazar, Marianela |  |  |
| **Cuba** | | |
| Infante, Mirta |  |  |
| **Ecuador** | | |
| Carrera Estupiñán, Enrique |  |  |
| Mora, Javier |  |  |
| **El Salvador** | | |
| Valdez, Marisabel |  |  |
| Moreno, Javier |  |  |
| **México** | | |
| Velarde, Antonio |  |  |
| Mayorga, Tania |  |  |
| Castro-Narro, Graciela |  |  |
| **Panamá** | | |
| Mayo, Miguel Antonio |  |  |
| Enrique, Adames |  |  |
| **Paraguay** | | |
| Girala, Marcos |  |  |
| **Perú** | | |
| Garavito, Jorge |  |  |
| Rodríguez Romero, Kriss |  |  |
| Rocío Galloso |  |  |
| **Puerto Rico** | | |
| Rodriguez, Federico |  |  |
| **República Dominicana** | | |
| Perelló, Christie |  |  |
| Contreras, Fernando |  |  |
| **Venezuela** | | |
| Dagher, Lucy |  |  |
| **Uruguay** | | |
| Hernandez, Nelia |  |  |
| Mainardi, Victoria |  |  |
